# A Novel Plant-Based Protein Has Similar Effects Compared to Whey Protein on Body Composition, Strength, Power, and Aerobic Performance in Professional and Semi-Professional Futsal Players

**DOI:** 10.3389/fnut.2022.934438

**Published:** 2022-07-19

**Authors:** Filipe J. Teixeira, Catarina N. Matias, João Faleiro, Rita Giro, Joana Pires, Helena Figueiredo, Raquel Carvalhinho, Cristina P. Monteiro, Joana F. Reis, Maria J. Valamatos, Vítor H. Teixeira, Brad J. Schoenfeld

**Affiliations:** ^1^Bettery Lifelab, Bettery S.A., Lisboa, Portugal; ^2^Atlântica, Instituto Universitário, Fábrica da Pólvora de Barcarena, Barcarena, Portugal; ^3^Interdisciplinary Center for the Study of Human Performance, Universidade de Lisboa, Cruz-Quebrada, Portugal; ^4^Centro de Investigação em Desporto, Educação Física, Exercício e Saúde, Universidade Lusófona, Lisbon, Portugal; ^5^Athletic Club Oulu Football Club, Oulu, Finland; ^6^Grupo de Ativistas em Tratamentos, Lisboa, Portugal; ^7^José de Mello Saúde, Lisboa, Portugal; ^8^Departamento de Saúde do Futebol Clube do Porto, Porto, Portugal; ^9^Laboratory of Physiology and Biochemistry of Exercise, Faculdade de Motricidade Humana, Universidade de Lisboa, Cruz-Quebrada, Portugal; ^10^Neuromuscular Research Lab, Faculdade de Motricidade Humana, Universidade de Lisboa, Estrada da Costa, Cruz-Quebrada, Portugal; ^11^Faculty of Nutrition and Food Sciences, University of Porto, Porto, Portugal; ^12^Research Centre in Physical Activity, Health and Leisure (CIAFEL), Faculty of Sports, University of Porto, Porto, Portugal; ^13^Laboratory for Integrative and Translational Research in Population Health (ITR), Porto, Portugal; ^14^Futebol Clube do Porto, Porto, Portugal; ^15^Health Sciences Department, Lehman College, City University of New York, Bronx, NY, United States

**Keywords:** supplementation, protein, athletes, lean body mass, power, aerobic capacity

## Abstract

**Introduction:**

The effects of dietary protein on body composition and physical performance seemingly depend on the essential amino acid profile of the given protein source, although controversy exists about whether animal protein sources may possess additional anabolic properties to plant-based protein sources.

**Purpose:**

To compare the effects of a novel plant-based protein matrix and whey protein supplementation on body composition, strength, power, and endurance performance of trained futsal players.

**Methods:**

Fifty male futsal players were followed during 8 weeks of supplementation, with 40 completing the study either with plant-based protein (*N* = 20) or whey protein (*N* = 20). The following measures were assessed: bone mineral content, lean body mass, and fat mass; muscle thickness of the rectus femoris; total body water; blood glucose, hematocrit, C-reactive protein, aspartate aminotransferase, alanine aminotransferase, creatine kinase, creatinine, and estimated glomerular filtration rate; salivary cortisol; maximal strength and 1-RM testing of the back squat and bench press exercises; muscle power and countermovement jump; VO_2max_ and maximal aerobic speed. Subjects were asked to maintain regular dietary habits and record dietary intake every 4 weeks through 3-day food records.

**Results:**

No differences in any variable were observed between groups at baseline or pre- to post-intervention. Moreover, no time^*^group interaction was observed in any of the studied variables, and a time effect was only observed regarding fat mass reduction.

**Conclusions:**

Supplementing with either a novel plant-based protein matrix or whey protein did not affect any of the variables assessed in high-level futsal players over 8 wks. These results suggest that whey protein does not possess any unique anabolic properties over and above those of plant-based proteins when equated to an essential amino acid profile in the population studied. Furthermore, when consuming a daily protein intake >1.6 g/kg BW.day^−1^, additional protein supplementation does not affect body composition or performance in trained futsal players, regardless of protein type/source.

## Introduction

Futsal is a demanding team sport involving strenuous high-intensity bouts of running accelerations and decelerations along with kicking, tackling, turning, changes of direction, and repeated sprinting (1, 2). Although similar to football (soccer), futsal has different features i.e., unlimited number of substitutions, use of a smaller ball with less bounce, lower number of players (only four outfield players and one goalkeeper), smaller goals (with 2 ×3 m), shorter match duration (two equal periods of only 20 min with clock stoppage for fouls, etc.) (3, 4). Due to these specific features, research indicates that high-level futsal players require high levels of agility (5), muscle power (5–7), repeated sprint ability (2, 8, 9), jumping (10), and aerobic performance (3, 11). In fact, 5 to 10% of the distance covered during a futsal match is performed while sprinting (12–14), which when associated with other tasks (i.e., kicking, turning, etc.) requires high strength and power development of the lower limbs (15). Additionally, like other small-sided games, high level futsal requires elevated levels of aerobic capacity (VO_2_max 55.2–62.8 ml.kg^−1^.min^−1^) (3).

Despite its growing popularity (12), research studies regarding dietary supplements in futsal athletes are scant. While evidence indicates that protein supplementation enhances muscle strength and hypertrophy, it may also play a role in muscle repair and recovery from endurance exercise (16), and further improve VO_2_peak with endurance training (17). Current protein recommendations for endurance athletes and those engaged in team sports range from 1.2 to 2.0 g/kg BW.day^−1^ (18–20), with earlier recommendations for football (soccer) ranging between 1.4 and 1.7 g.kg BW.day^−1^ (21) and more recent recommendations indicating potential benefits from higher amounts 1.6–2.2 g/kg BW.day^−1^ (22).

To optimize muscle hypertrophy in conjunction with regimented resistance exercise training (RET), a protein should provide 6–15 g of essential amino acids per serving (23, 24), including 1.7–3.5 g of leucine (24, 25), with a total daily protein intake of ~1.6 g/kg BW.day^−1^; timing, dose, and protein source appear to play only a minor role in the process (16). Several research studies have compared whey protein with plant-based proteins such as rice (26, 27), pea (28), and soy (29, 30), with no differences in body composition or strength observed between groups. Conversely, other research has found superior results with whey or milk protein when compared to soy (31, 32). No research has been performed comparing a plant-based protein to whey regarding team sports, i.e., futsal. Thus, the purpose of this study was to compare supplementation with whey vs. a protein matrix combining multiple plant-based protein sources fortified with branched-chain amino acids (BCAA) (33) on measures of body composition, strength, power, and aerobic performance in futsal players. Given that both protein sources provided identical amounts of protein and essential amino acids, we hypothesized that changes would be similar between conditions.

## Methods

### Ethics

This investigation was approved by the Faculty of Human Kinetics Institutional Review Board (approval number 37/2021) and conformed to all standards of human research set out in the declaration of Helsinki (34). The trial was registered at clinicaltrials.gov as NCT05228236 (https://clinicaltrials.gov/ct2/show/NCT05228236). Prior to engaging in any of the study procedures, the purpose and design of the study, the data collection methodologies, and all potential risks and benefits were explained to potential research participants. All participants gave their verbal and written informed consent before enrolling.

### Participants

**Figure 1:**
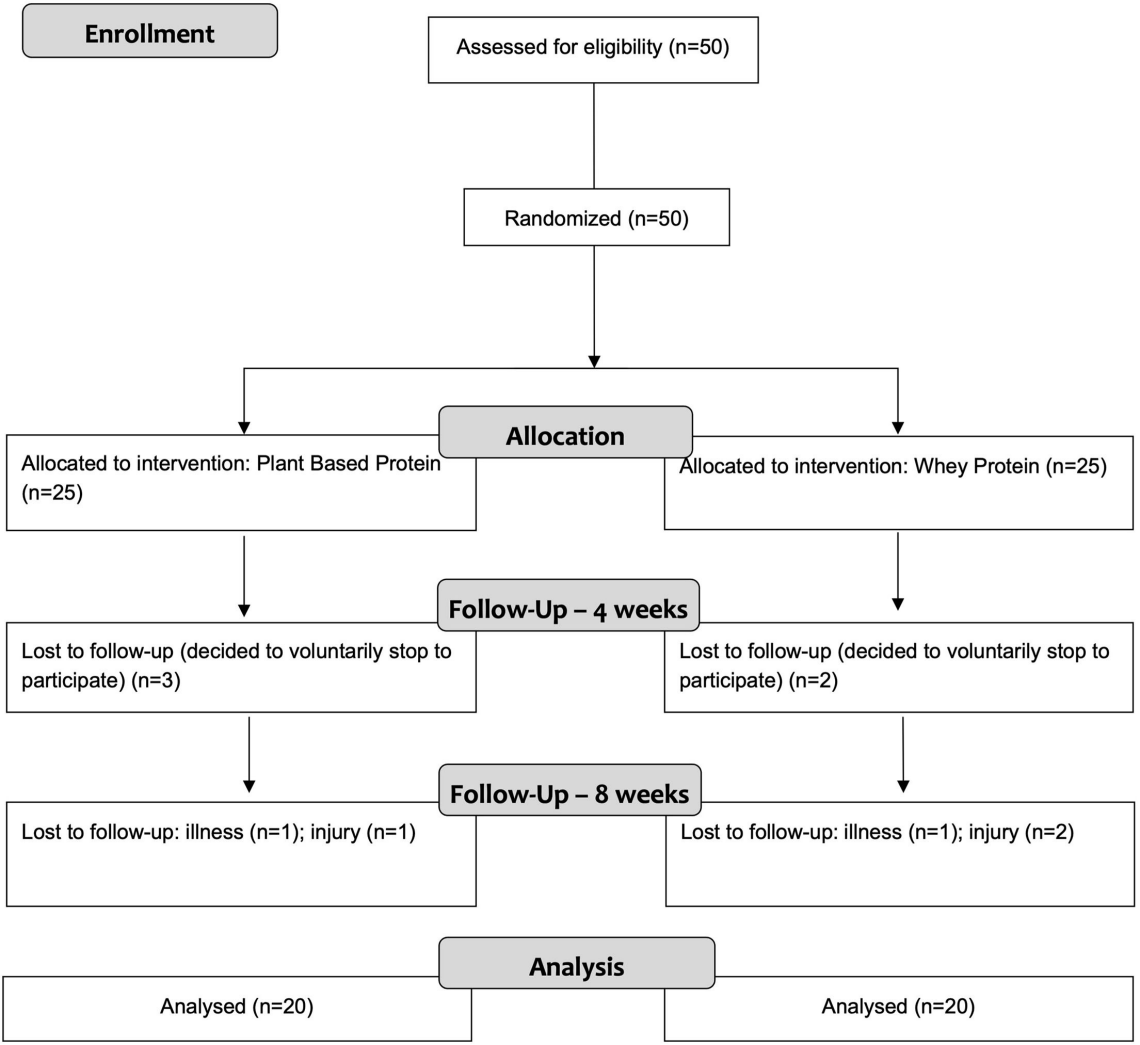
CONSORT diagram of the randomization and flow of participants through the study.

Fifty male futsal players, not engaged in RET, at the time of the investigation, volunteered to participate in this 8-week study. From the initial sample, forty completed the investigation. Participants were between the ages of 18 and 35 y and were recruited from national level futsal sports clubs. They were randomly assigned to one of two groups: novel plant-based protein (PB) or whey protein (WP). For details, please refer to the CONSORT flow diagram (Figure 1). Baseline measures were performed pre-season while wk 4 and 8 were assessed during the competitive season, in the first half of the regular phase of the championship.

The final sample was comprised of both professional and semi-professional players. The professional players (*n* = 16) participated in the Major Portuguese National Futsal League “LIGA PLACARD” and had a sport-specific training frequency of 5 times per week (5×/wk) and a weekend game frequency of 1×/wk. Each training session lasted ~1.5 h.

The semi-professional players (*n* = 34) participated in either the second (*n* = 17) or third (*n* = 17) Portuguese National Futsal League. Training sessions lasted ~1.5 h for both teams and athletes had a training frequency of 3×/wk and a weekend game frequency of 1×/wk.

Participants represented the top five teams of each division and included players from the current and former national team. Although the players were from different teams, they had similar training schemes, where sport-specific training regimen was normally comprised of mobility and injury prevention exercises for ~20 min, followed by technical and tactical components for ~30 min. The remainder of the training session was dedicated to open exercises, mimicking game conditions. RET was not part of the coaches' training prescription at the initial phase of the season, nevertheless, three players reported that they occasionally went to the gym on their own to perform RET.

#### Sample Size and Study Design

Most previous research comparing plant-based whey proteins show similar effects on body composition and performance (26–28, 35), assuming equal amounts of protein and essential amino acids are provided. Anticipating similar results and bearing in mind that when there are no differences between the arms of the study, a significant effect size does not exist, we employed a non-inferiority trial. Nevertheless, the only study that found significant differences between a plant-based protein (soy) and whey protein reported an 80% power at an α-level of 5%, with a 0.5 kg difference in lean body mass (LBM) (32). Assuming similar conditions but 85% power, an alpha of 0.05, and a 0.5 effect size (36), GPower (version 3.1, Dusseldorf, Germany) calculated a required sample of 36 participants. Considering a 25% dropout, a sample of 48 participants would be required and we recruited 50 participants as a buffer against attrition.

#### Supplementation and Randomization Protocol

All protein powders presented a third-party certificate of analysis regarding their nutritional composition (Table 1) and were tested for doping-controlled substances (LGC group, Middlesex, UK). Additionally, the protein content of both supplements was re-assessed at our laboratory using a Dumatherm automatic Nitrogen analyzer (C. Gerhardt GmbH, Germany) according to the manufacturer's guidelines. More specifically, a sample within 90–140 mg was weighted in an aluminum foil (Dumafoil, C. Gerhardt GmbH, Germany) using an analytical scale (Kern model ABS 320-4N, GmbH, Germany). The sample was then compacted and inserted into the device. The results were analyzed considering a conversion factor of 6.25 (for PB) and 6.38 (for WP) per N gram, an O_2_ factor of 1.8 mL/mg and a 300 ml/min of O_2_ flow rate.

**Table 1:** Supplementation nutritional composition.

	**PB**	**WP**
Dose per serving (g)	32	34
Energy (kcal)	130	135
Fat (g)	2.7	2.4
Saturated fats	0.6	1.5
Carbohydrates (g)	0.6	2.7
Sugars	<0.5	2.1
Protein (g)	25	24.8
**Essential amino acids (g)**		
Histidine	0.6	0.4
Isoleucine^*^	1.1	1.4
Leucine^*^	2.2	2.4
Lysine	1.7	1.8
Methionine	0.3	0.3
Phenylalanine	1.1	0.8
Threonine	0.9	1.2
Tryptophan	0.2	0.1
Valine^*^	1.1	1.0
∑ Essential Amino acids (g)	9.2	9.4
∑ BCAA^*^ (g)	4.4	4.8

* *BCAA, branched-chain amino acids; PB, plant-based protein; WP, whey protein*.

The PB supplement was comprised of a proprietary blend (BETTPRO^®^) containing pea protein isolate (85% PRO concentration), yeast protein (80% PRO concentration), and BCAA 4:1:1, currently, patent-pending (Bettery S.A., Oeiras, Portugal), while the WP supplement was comprised of a whey protein concentrate yielding an 80% protein concentration (Ewalco AB, Gothenburg, Sweden). The proprietary blend was mostly comprised of pea protein (>70%) with the yeast and BCAA included to complement and match the essential amino acid profile of whey. Both supplements required the addition of fat-reduced cocoa powder, emulsifiers, thickeners, and sweeteners to ensure similar taste and texture. The PB required the use of masking excipients to neutralize the typical flavor of the pea protein. Both supplements were weighted in single-dose (see Table 1 for supplement composition) individual plastic bags using a digital scale model EMB 5200 G 5 (Kern, Balingen, Germany). The participants were instructed to dissolve the content of each bag within 250 mL of water. A 2-week supply was delivered to each player; a researcher collected the empty plastic bags to assess adherence while delivering a new supply. Participants received the packaged supplements and instructions in a double-blinded fashion. Supplementation compliance (a minimum of 90% compliance was deemed acceptable), possible side effects, and subjectively perceived fatigue and muscle recovery were assessed by questionnaire at the end of each 4-week block of supplementation.

Participants were randomized, within each competitive level, using a covariate adaptative randomization method based on age, grip strength, DXA-measured fat (FM), and LBM. Accordingly, at baseline, there were no statistically significant differences between groups regarding all the reported variables (Tables 2, 3). The investigator responsible for the sample randomization and supplement distribution was not directly involved in participants' eligibility interviews or data collection. All other investigators and all the participants were blind to the supplementation conditions and randomization sequence.

**Table 2:** Energy and macronutrient intake at baseline and after 8 weeks of intervention.

	**PB (*n* = 20)**			**WP (*n* = 20)**		
	**Baseline**	**Post 4 weeks**	**Post 8 weeks**	**Baseline**	**Post 4 weeks**	**Post 8 weeks**
Energy (kcal)	2,057.3 ± 350.3	2,073.5 ± 309.9	2,064.2 ± 366.7	2,055.5 ± 242.1	2,093.5 ± 246.3	2,080.5 ± 255.0
Energy (kcal/kg FFM)	36.3 ± 8.1	36.1 ± 9.5	35.8 ± 7.1	36.1 ± 6.7	36.2 ± 6.0	37.1 ± 6.4
Protein (g)	113.6 ± 26.6	117.7 ± 21.5	121.1 ± 21.4	118.2 ± 27.2	118.6 ± 28.0	121.7 ± 20.9
Protein (g/kg BW.day^−1^)	1.6 ± 0.5	1.7 ± 0.2	1.7 ± 0.3	1.6 ± 0.5	1.7 ± 0.4	1.7 ± 0.4
Fat (g)	84.9 ± 17.4	84.6 ± 18.8	84.1 ± 16.3	85.5 ± 22.5	83.2 ± 15.0	84.2 ± 18.4
Carbohydrate (g)	203.9 ± 51.9	202.7 ± 54.4	198.8 ± 58.0	188.5 ± 32.9	201.5 ± 33.8	200.8 ± 38.2

*BW, body weight; PB, plant-based protein; WP, whey protein*.

**Table 3:** Body composition, performance, and hematological and biochemical markers at baseline and after 4 and 8 weeks of intervention.

	**PB (*n* = 20)**			**WP (*n* = 20)**		
	**Baseline**	**Post 4 weeks**	**Post 8 weeks**	**Baseline**	**Post 4 weeks**	**Post 8 weeks**
**Body composition**						
Body mass (kg)	70.8 ± 9.2	70.5 ± 9.3	69.9 ± 9.0	71.6 ± 10.2	70.6 ± 7.7	69.7 ± 7.7
TBW (L)	43.1 ± 4 4	43.0 ± 4.3	42.9 ± 4.4	43.7 ± 4.3	43.1 ± 3.7	42.8 ± 4.0
Muscle thickness rectus femoris (mm)	26.7 ± 3 6	27.2 ± 2.7	28.0 ± 3.2	26.4 ± 3.4	27.0 ± 2.9	27.9 ± 2.7
Bone mineral content (kg)	2.93 ± 0.39	2.96 ± 0.38	2.97 ± 0.43	3.06 ± 0.45	3.06 ± 0.46	3.07 ± 0.42
Lean body mass (kg)	57.7 ± 5.8	57.9 ± 5.4	58.5 ± 6.3	58.0 ± 5.9	58.2 ± 5.3	58.9 ± 5.5
Lean soft tissue (kg)	54.6 ± 5.5	55.0 ± 5.1	55.3 ± 5.9	54.8 ± 5.5	55.1 ± 4.97	55.7 ± 5.1
Fat mass (kg)	13.1 ± 5.0	12.1 ± 5.0	11.4 ± 4.1^#^	13.6 ± 5.4	11.5 ± 3.5	10.9 ± 3.2^#^
Fat mass (%)	18.1 ± 4.9	17.6 ± 4.8	16.1 ± 4.2	18.5 ± 4.8	17.7 ± 3.7	15.4 ± 3.2
Visceral fat area (cm)	61.9 ± 21.5	59.6 ± 20.0	57.1 ± 21.3	57.1 ± 18.3	53.1 ± 11.9	51.2 ± 10.4
**Muscle strength**						
Handgrip dominant hand (N)	471.2 ± 91.2	483.4 ± 93.8	486.4 ± 89.3	461.3 ± 75.9	470.5 ± 74.0	475.4 ± 73.7
Back squat 1 RM (kg)	76.6 ± 14.3	78.4 ± 13.8	85.5 ± 15.6	80.2 ± 14.7	81.0 ± 13.8	85.9 ± 13.1
Bench press 1 RM (kg)	54.6 ± 13.0	55.3 ± 16.2	56.9 ± 9.1	56.8 ± 8.6	57.4 ± 9.4	59.1 ± 9.7
Counter movement jump (cm)	33.5 ± 4.2	34.7 ± 4.5	38.0 ± 4.7	33.5 ± 3.9	36.9 ± 4.0	38.7 ± 3.4
**Anaerobic and aerobic performance**						
Anaerobic peak power (W/kg)	12.9 ± 4.50	–	11.4 ± 3.2	12.4± 4.8	–	11.5 ± 4.2
Anaerobic average power (W/kg)	8.5 ± 1.0	–	8.4 ± 0.7	8.3 ± 0.9	–	8.1 ± 0.9
Anaerobic power drop (%)	62.7 ± 15.4	–	58.5 ± 11.1	64.0 ± 16.9	–	62.8 ± 14.0
VO_2max_ (mL/kg/min)	49.7 ± 7.1	–	50.6 ± 7.6	51.2 ± 7.0	–	51.1 ± 3.3
VO_2max_ (mL/min)	3,545.8 ± 436.8	–	3,544.8 ± 380.9	3,719.1 ± 410.8	–	3,582.7 ± 294.8
MAS (km/h)	16.0 ± 1.6	–	16.3 ± 1.9	15.0 ±1.9	–	16.2 ± 1.2
**Hematological and biochemical markers**						
C Reactive protein (mg/L)	10.0 ± 5.0	–	9.4 ± 1.2	8.9 ± 2.2	–	8.3 ± 0.7
Hematocrit (%)	44.2 ± 3.3	–	43.6 ± 2.6	44.4 ± 2.4	–	44.3 ± 2.4
Creatine kinase (U/L)	254.4 ± 126.3	–	201.0 ± 68.9	272.5 ± 114.1	–	195.5 ± 55.6
Alanine aminotransferase (U/L)	22.8 ± 15.4	–	19.0 ± 6.1	21.0 ± 9.6	–	20.2 ± 6.9
Aspartate aminotransferase (U/L)	25.3 ± 16.9	–	20.5 ± 6.9	19.3 ± 7.4	–	19.7 ± 5.9
Glucose (mg/dL)	77.8 ± 13.1	–	70.1 ± 11.6	76.6 ± 13.6	–	67.6 ± 12.4
Creatinine (mg/dL)	1.4 ± 0.2	–	1.5 ± 0.2	1.4 ± 0.3	–	1.5 ± 0.2
Estimated glomerular filtration rate (mL/min)	82.1 ± 17.3	–	80.8 ± 12.6	83.4 ± 20.2	–	79.6 ± 20.1
Salivary cortisol (μg/dL)	1.2 ± 0.6	–	1.2 ± 0.4	0.9 ± 0.6	–	0.7 ± 0.5

*PB, plant-based protein; WP, whey protein; TBW, Total body water; MAS, maximal aerobic speed*.

# *Time effect from baseline (ANCOVA)*.

*Results are presented as mean ± SD*.

### Assessments

Athletes' anthropometry, body composition, strength, power, aerobic performance, nutritional intake, health, and safety biochemical parameters were assessed at baseline and week 8. A partial evaluation was performed in week 4 that included anthropometry, body composition, strength, power, and nutritional intake assessment. All evaluations of the participants, including blood and saliva samples collection, were performed in our laboratory facilities, early in the morning (7 a.m.) after a 12-h fast and without consumption of alcohol, caffeine/stimulant beverages, and at least 12 h from the last exercise session. Biofluids and body composition assessments were performed fasting, while the performance evaluations were made in a fed state, with the consumption of a meal replacement bar (nutritional composition: 231 kcal, 14 g fat, 12 g carbohydrate, and 13 g protein).

#### Anthropometry

Participants had their weight and height measured wearing minimal clothing and without shoes to the nearest 0.1 kg and 0.1 cm, respectively, with a scale and a stadiometer (Seca, Hamburg, Germany) using standardized procedures as reported elsewhere (37).

#### Body Composition

Body composition was determined by three methodologies:

(a) Dual energy X-ray absorptiometry (DXA) (Horizon Wi, Hologic, Waltham, USA) where participants underwent a whole-body DXA scan according to the procedures recommended by the manufacturer. The same technician positioned the patient, performed the scan, and executed the analyses. The DXA measurements included whole-body measurements of bone mineral content (BMC, g), LBM (kg), as well as absolute and percentage fat mass (FM, kg, and %). Within FM, visceral adipose tissue was distinguished using DXA software (38).

(b) B-mode ultrasonography for muscle thickness (MT) measurement of the rectus femoris (RF) (39) using a 7.5 MHz linear-array transducer (model WED-180 HL, Welld, Shenzhen, China). Longitudinal and transversal scans were obtained at the muscles' mid-belly at 56% of the distance from the proximal edge of the patella to the anterior superior iliac spine (40). Participants were positioned in a seated position with their knees flexed at 90° (0° being a full extension), participants' legs were supported during the scan and their muscles relaxed. To ensure that repeated scans (weeks 4 and 8) were taken from the same site, scanning locations were mapped with a malleable transparent plastic sheet at the baseline measurement, along with other distinguishing surface landmarks (e.g., border of the patella, tattoos, scars, moles). We defined MT as the perpendicular distance between the subcutaneous adipose tissue-muscle interface and intermuscular interface. Averaged values from three measurements were considered for further analysis. All measures were collected and digitally analyzed by the same operator who was blinded to group allocation. The test-retest CV in 29 participants for the MT in RF using ultrasonography in our laboratory is 2.1%.

(c) Bioelectrical impedance analysis (BIA) is a phase sensitive device, from which whole-body resistance (R) and reactance (Xc) are obtained using a single frequency of 50 kHz (BIA 101 BIVA^®^PRO, Akern S.R.L., Pisa, Italy). Device calibration was performed according to the procedures recommended by the manufacturer. Assessments were obtained after a 10-min rest period in a supine position following the guidelines for athletes stated elsewhere (41). From the raw data R and Xc, total body water (TBW) was determined using Akern Software (version 1.19.2). The test-retest CV in 15 participants for R and Xc in our laboratory is 3.5 and 1.5%, respectively.

#### Hematological and Biochemical Analysis

Saliva and whole blood were collected into salivettes and EDTA tubes by standard procedures. Blood measurements included the assessment of glucose, hematocrit (Hct), C-reactive protein (CRP), aspartate aminotransferase (AST), alanine aminotransferase (ALT), creatine kinase (CK), and creatinine, using photometry techniques in automated equipment (Vario Photometer II DP310, Diaglobal Gmbh, Berlin, Germany; Nycocard reader II, Abbott, Chicago, Illinois, EUA). Creatinine values were used to calculate the estimated glomerular filtration rate (eGFR) (42). Cortisol was evaluated in saliva through Enzyme-Linked Immunosorbent Assay (ELISA) commercial kits (Salimetrics, PA, USA) in an automated reader (800 TS Absorbance Reader, Biotek, Vermont, USA).

#### Performance Tests

##### Muscle Strength

Maximal isometric handgrip strength of the dominant hand was determined using a Jamar^®^ hydraulic hand dynamometer (Jamar, Sammons Preston, Inc, Bolingbrook, IL). The participants were tested in a standing position with an elbow in full extension (43). The participant was asked to squeeze the dynamometer at maximal effort for three trials, with a 30-s break between each trial. The best of the three trials was considered for data analysis.

Maximum strength (1-RM) was predicted based on load-velocity relationship for the back squat and bench press exercises on a Multipower machine. For both back squat and bench press, participants performed one set of 8 repetitions at a self-selected load, having been instructed to perform the concentric phase as fast as possible. A linear encoder (Vitruve Encoder, Madrid, Spain) was attached to the barbell during the tests, measuring its vertical displacement and velocity. The higher mean propulsive velocity along the 8 repetitions was used to predict the 1-RM (44). The determination of 1RM was directly supervised by an NSCA-certified strength and conditioning specialist.

Jump height was assessed by a countermovement jump which was performed on a contact-mat jump system controlled by an open-source hardware and software model (Chronojump, Barcelona, Spain). The displacement of center of gravity (jump height h) during the flight was estimated by means of flight time (t) through a standardized kinematic equation h = t2.g/8, where g is gravity (45). The best attempt out of three was considered for analysis.

Participants were familiarized with the muscle strength tests prior to our investigation.

##### Anaerobic and Aerobic Performance

Anaerobic power performance was assessed *via* a supramaximal cycling test—Wingate, performed on a cycle ergometer (Monark ergomedic 894 E, Monark Exercise AB, Vansbro, Sweden). Participants were instructed to cycle as fast as possible against a predetermined resistance (7.5% of the participant's body mass)for 30 s (46). Variables collected at the end of the test included anaerobic peak power, anaerobic average power, and anaerobic drop power, calculated as the percentage drop between peak and minimum power of the test.

Aerobic performance was assessed *via* VO_2max_ and maximal aerobic speed (MAS), determined by a breath-by-breath gas analyzer (Quark, Cosmed, Italy) in an incremental treadmill test. After a 3-min warm-up at 5 km·h^−1^, participants began the test at 6 km·h^−1^ and 2% grade. Each minute the speed increased 1 km·h^−1^ until volitional exhaustion so that fatigue would be induced within 8–12 min (47). Standardized verbal encouragement was given throughout the test. The MAS was considered as the speed of the last stage completed (48) while VO_2max_ was considered the highest 30 s average value of VO_2_ and assumed when at least 2 of the following criteria were met: respiratory quotient >1.10; heart rate (HR) equal to or >95% of predicted maximal HR (calculated as 208–0.7 × age); and increments in VO_2_ below 2 ml·kg^−1^·min^−1^ despite an increase in speed (49).

#### Diet Control and Supplementation

Three-day food records (2 weekdays and 1 weekend day) were requested to characterize the food intake of the participants at baseline, weeks 4 and 8. Individuals were instructed to maintain their normal dietary intake during the 8-week study period. Food records were then analyzed by software [Nutritics Research Edition (v5.09), Dublin, Ireland] for total energy and macronutrient consumption, by a registered dietitian.

After evaluations, each participant received either PB or WP. Participants were only aware that these were protein supplements and that compounds were distributed in a double-blinded manner. Both groups consumed the protein supplements 30–60 min after exercise on training days and 30–60 min before bedtime on non-training days.

### Statistical Analysis

IBM SPSS Statistics version 25.0, 2012 (IBM, Chicago, Illinois, USA) was used to analyze the data. Basic descriptive statistics were run to characterize the study participants. All variables were assessed for normality using the Kolmogorov–Smirnov test. Independent sample *T*-tests were used to compare means between groups at baseline. *Post-hoc* intention to treat (ITT) analysis was applied to investigate the effects of intervention over time within and between groups through generalized estimated equations (GEE) analyses. Time and time-by-group interactions were analyzed by analysis of covariance (ANCOVA), using baseline measurements as the covariate. The equality of the matrix of variance and sphericity were explored with the Levene F-test and Mauchly's test, respectively. Significance for α was set at *p* ≤ 0.05.

## Results

Data from 40 participants were considered for final analysis (PB = 20, WP=20). Sample losses were due to personal reasons (*N* = 5) or due to injury during practice (*N* = 5), therefore an intervention adherence of 80% was observed. No differences were observed between groups at baseline nor after 8 weeks of supplementation. Analysis of food records showed no differences between groups at baseline nor pre- to post-study regarding energy and macronutrient intake (Table 2).

Participants were compliant with the supplementation, taking 93 ± 1% of supplements with no adverse effects reported throughout the study. No differences were found between groups regarding general perceived side effects of the supplementation. Additionally, perceived gastrointestinal disturbances were 18% higher in the WP group.

**Figure 2:**
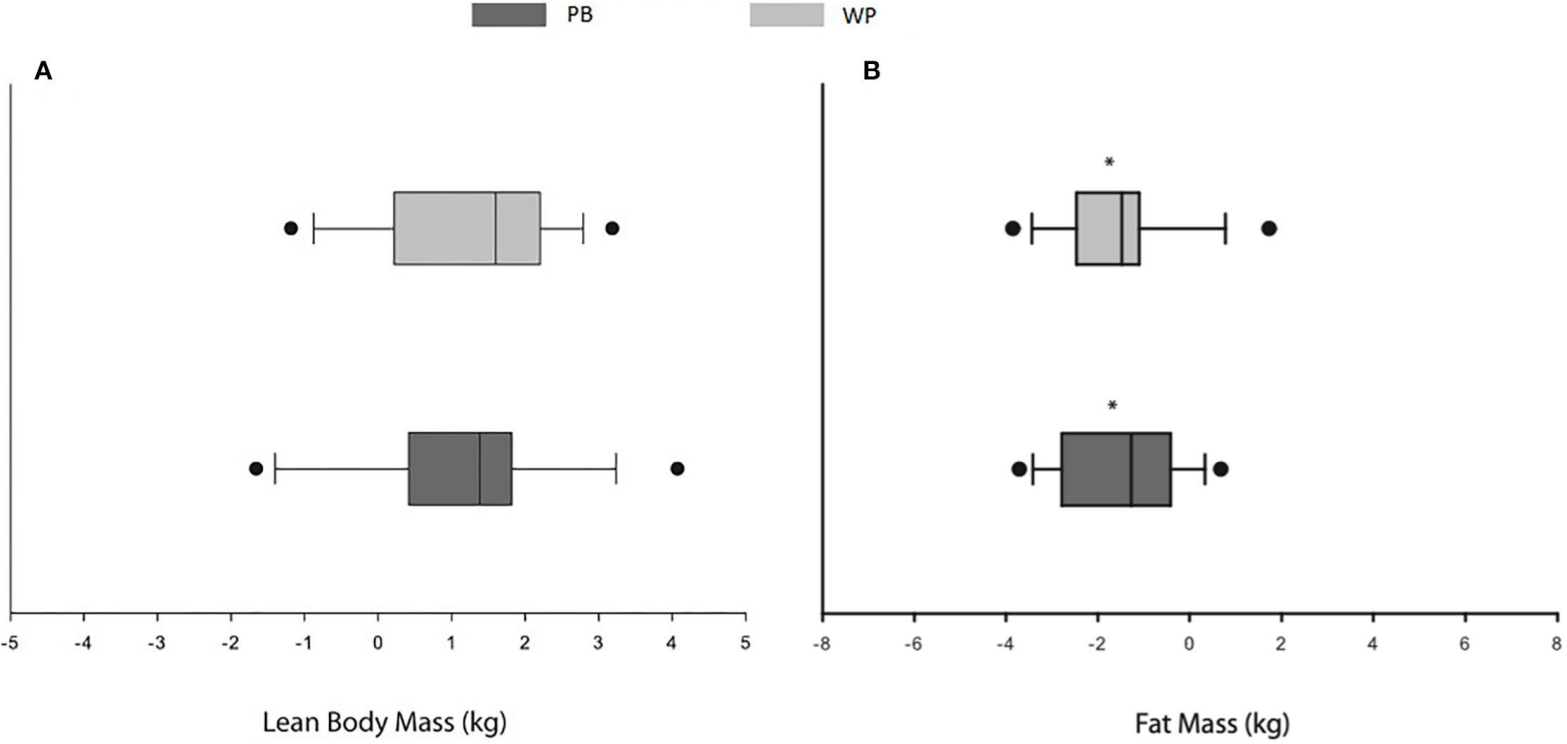
Changes (Δ baseline-week 8) in lean body mass **(A)**, fat mass **(B)** during the 8-week supplementation protocol. Data are shown as box and whisker plots where whiskers are the maximum and minimum and the box represents the interquartile range, the line the group median. Dots represent outliers. *Represents an observed time effect. PB, plant-based protein; WP, whey protein.

**Figure 3:**
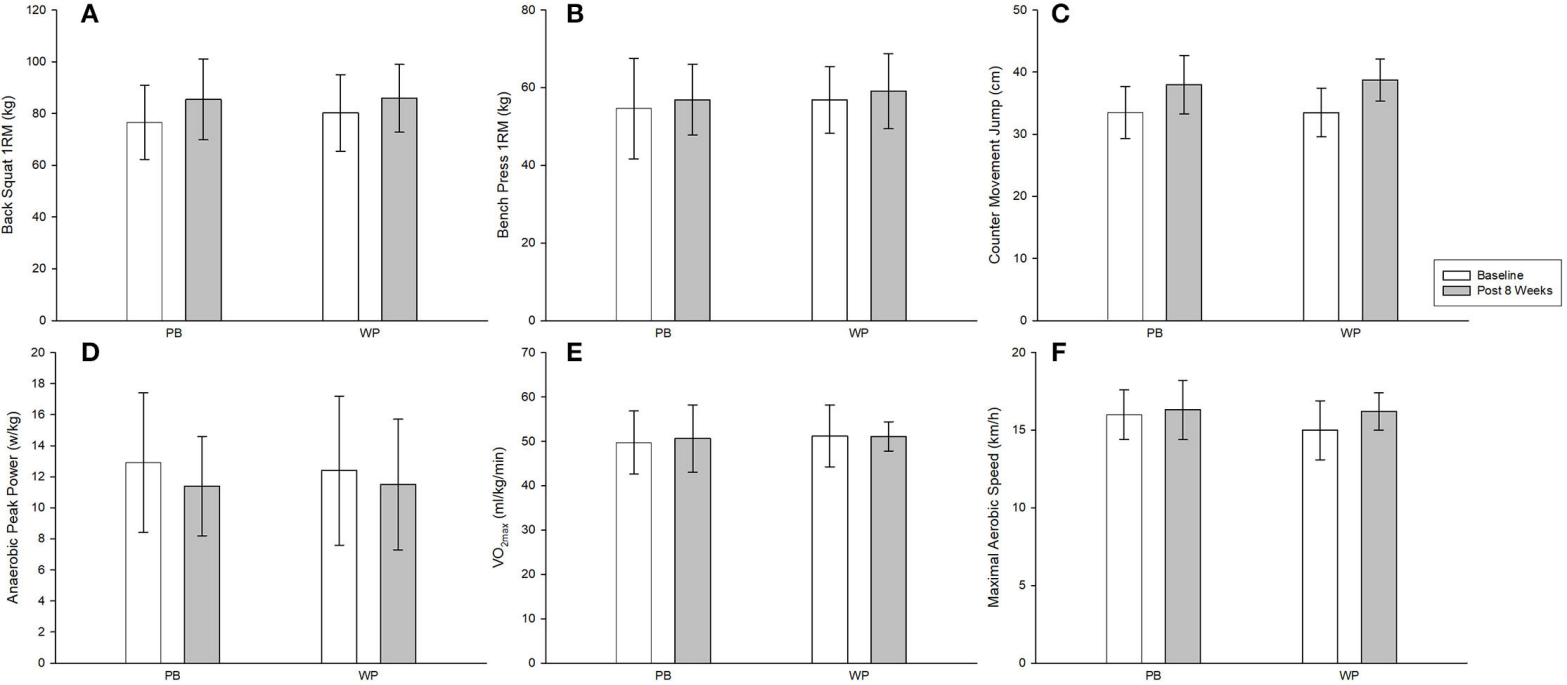
Performance markers at baseline and post 8 weeks: back squat **(A)**, bench press **(B)**, counter movement jump **(C)**, anaerobic peak power **(D)**, VO2max **(E)** and maximal aerobic speed **(F)**. PB, plant-based protein; WP, whey protein.

No differences were observed between conditions at baseline regarding all variables (see Table 3; Figures 2, 3). Moreover, no differences were observed between groups from baseline to week 8 (*p* > 0.05). No time^*^group interaction was observed in any of the studied variables, and a time effect was only observed for a reduction in FM. Similar results were observed when the analysis was carried out using ITT.

## Discussion

### Body Composition

We found no differences between groups regarding all body composition variables when comparing PB and WP in professional and semi-professional futsal athletes. These findings are in line with previous populations (26–30, 35) but not with others (31, 32, 50, 51). Of note, a study by Babault et al. (28) that compared pea protein with whey used 25 g 2 times a day (50 g daily) on untrained subjects. Compared to the present study, this investigation (28) was of longer duration (12 wks), used only three exercises (involving elbow flexor and extensor muscles), and involved a higher daily protein supplementation in untrained men. Moreover, Babault et al. (28) did not control the dietary intake and the pea protein supplement offered a lower amount of leucine per serving than in our study (1.6 vs. 2.2 g). Another study compared pea protein with whey protein, in trained subjects (35), performed a combination of high-intensity functional training (HIFT) and no differences were found between groups after 8 wks of HIFT, with only a time effect being reported regarding 1-RM squat and deadlift. Limitations in that study included a small sample size (*n* = 15) and the use of a BIA model with questionable accuracy (52, 53).

Studies from Joy et al. (26) and Moon et al. (27) that compared rice protein with whey protein also presented several limitations. As acknowledged by the authors, sample sizes were relatively small (*n* = 24) and the Moon et al. (27) work lacked training supervision. Moreover, neither study employed a direct measure of hypertrophy, which may have limited the ability to detect subtle longitudinal changes in muscle mass (54). The sample in our study was comprised of professional and semi-professional futsal players without RET exposure, which explains the absence of a time effect pertaining MT and fat free mass (FFM) gains, even when consuming at least 1.6 g/kg BW.day^−1^ of protein (16). Still, the FFM gains in both groups are in line with the values reported in some meta-analysis (16, 55) and with the works by Joy et al. (26) and Moon et al. (27) using rice protein. Our results pertaining to MT are also consistent with the work by Banazsek et al. (35), who found no differences between pea protein and whey protein after 8 wks of HIFT.

In contrast, Volek et al. (32) reported superior increases in FFM favoring WP vs. soy protein in untrained subjects. It should be noted that the supplements used by Volek et al. (32) also provided carbohydrates and the soy protein supplement provided a lower amount of leucine than the PB in our study (1.4 vs. 2.2 g). Furthermore, the amount of leucine provided from both supplements in Volek et al. (32) differed greatly (1.4 g of leucine in the soy protein group vs. 2.2 g in the WP), which is particularly important given the recommended leucine dosage for maximizing muscle protein synthesis (1.7–3.5 g per meal) (24, 25). Other studies that reported conflicting findings with ours did not compare isolated protein sources (50), and assessed dairy protein vs. soy protein in elderly participants (31).

In our study, a time effect was noted regarding FM loss, with no differences between groups. These results are in agreement with some studies (26, 27, 32) but not others (28, 35). This might be explained by the reduced energy availability in both arms of our study (PB: 35.8 ± 7.1; WP: 37.1 ± 6.4 kcal/kg FFM), which seems more appropriate for fat loss (56). Moreover, research indicates a higher protein intake might mitigate FFM loss during periods of energy restriction (57, 58), which might explain the FM reduction while maintaining FFM. Taken together, the absence of a RET protocol and the reduced energy availability in our trial might explain the lack of a time effect pertaining to FFM gains and the reduction of FM in both conditions. Furthermore, since the dietary intake did not change throughout the study, one might speculate that a natural increase pertaining to in-season training volume might have led to the reported FM reduction in both groups.

### Muscle Strength

No differences were found between groups regarding both muscle strength markers, nor did we observe a time effect. These results are in contrast with protein supplement research performed in combination with RET (26–28, 32, 35). Since our sample was comprised of futsal players not engaged in RET, these results are not surprising. A meta-analysis by Morton et al. (16), found that protein supplementation has little effect on strength when compared to RET when individuals consume >1.6 g/kg BW.day^−1^ of protein. Similar results have been reported regarding protein supplementation on muscle force production with both acute and chronic concurrent training (59). Still, when comparing our results with the study from Banazsek et al. (35), which used a sample closer to ours (HIFT), our results are in line with all findings except for 1-RM. It could be hypothesized that protein supplementation might improve performance *via* enhanced recovery. However, a recent study showed no differences between a high intake of pea protein or WP when compared with water intake on post-exercise delayed onset muscle soreness (60), casting doubt on the hypothesis.

### Anaerobic and Aerobic Performance

No between-group differences or time effects were found regarding anaerobic or aerobic performance. Few studies have investigated the effects of protein supplementation on anaerobic (59) and aerobic performance (61–63). Regarding protein supplementation on muscle power Camera et al. (59) showed similar results to ours. Also, it seems that when adequate amounts of carbohydrates are consumed, protein supplementation does not further increase aerobic performance (24). Moreover, our results agree with a recent systematic review showing that protein supplementation increases myofibrillar but not mitochondrial protein synthesis (64), consequently failing to enhance whole-body aerobic power (i.e., VO_2max_).

### Hematological and Biochemical Markers

No effects of time or group were detected regarding all hematological and biochemical markers. Only one previous study by Nieman et al. (60) compared pea protein vs. WP after a 90-min eccentric exercise bout regarding proxy markers of muscle damage (CK) in non-athletic, non-obese males. Results showed that WP delayed the elevation of CK to a greater extent than pea protein or water. These results conflict with ours since we did not find any differences between groups. Discrepancies in findings can potentially be explained by the fact that we studied a different population (trained futsal players) and did not include a specific muscle damage-inducing protocol. Moreover, we used a novel protein/BCAA matrix that combined not only pea protein but also yeast protein and BCAA as well, yielding an equal amino acid profile to whey protein.

Regarding ALT and AST (proxy markers of liver health) our null findings align with those of Nieman et al. (60). The lack of changes in eGFR and creatinine in our study is consistent with a plethora of research showing that relatively high protein diets do not have negative effects on renal health in healthy individuals (65–67) or even in overweight/obese individuals with mild kidney function impairment (68). In brief, both proteins displayed a good safety profile after 8 wks of supplementation, with no changes in inflammatory markers (CRP), red blood cell profile (Hct), liver (ALT/AST), glucose or kidney function (creatinine, eGFR), being observed between groups.

### Strengths and Limitations

This is the first study to compare a plant-based protein vs. whey in professional and semi-professional team sports athletes (i.e., futsal). Few (or no) studies have compared the effects of a protein source when provided as supplemental protein outside RET or strength/power sports (61–63), with only one comparative study being performed in collegiate female basketball players (whey vs. casein) (69). Furthermore, we supplemented with an iso-energetic, iso-nitrogenous, iso-EAA novel plant-based protein/BCAA mixture that matched whey protein, using yeast protein for the first time. Moreover, we assessed not only body composition using multiple gold standard methods but also anaerobic and aerobic performance, as well as several hematological and biochemical markers, thus providing a complete longitudinal view of the effects of both proteins. While this study provides novel insights, it nevertheless presents some limitations. For one, the inclusion of a placebo/control group would have added important information to the findings. In addition, we did not assess the effectiveness of the blinding and thus cannot be completely sure that there was not a residual placebo effect. Additionally, the participants spontaneously reduced their protein intake from other sources, when supplementing with either protein. Albeit not interfering with their usual diet adds ecological validity to our study, it precludes any conclusions from an increase in dietary protein. Moreover, extending the study length to 12 wks or more and employing a RET protocol in both groups would have provided additional insights into the effects of the proteins on measures of strength and body composition. It is also important to bear in mind that DXA, as well as BIA-derived parameters, have different instrumental sensitivities thus not allowing for direct comparisons between studies that measure R, Xc, and/or water compartments with different technologies or sampling frequencies (70, 71). As previously discussed, although not changing the diet of the participants adds ecological validity to this study, this might have influenced some performance outcomes (i.e., aerobic capacity) due to reduced energy and carbohydrate intake.

## Conclusion

In summary, supplementing with either a novel plant-based protein matrix or whey protein did not affect any of the variables assessed in high-level futsal players over 8 wks. These results suggest that whey protein does not possess any unique anabolic properties over and above those of plant-based proteins when equated for essential amino acid profile in the studied population. Furthermore, when consuming a daily protein intake >1.6 g/kg BW.day^−1^, additional protein supplementation does not affect body composition or performance in trained futsal players, regardless of protein type/source.

## Data Availability Statement

The datasets presented in this article are not readily available because the portuguese data protection law does not allow to share data of the participants. The database can only be accessed by the investigation team. Requests to access the datasets should be directed to filipe.teixeira@betterylife.com.

## Ethics Statement

The studies involving human participants were reviewed and approved by Faculty of Human Kinetics Institutional Review Board. The patients/participants provided their written informed consent to participate in this study.

## Author Contributions

FJT, CNM, CPM, JFR, MJV, and VHT: substantial contributions to the conception and design of the work. FJT, CNM, JF, RG, JP, HF, RC, and BJS: acquisition, analysis, and/or interpretation of data for the work. BJS: statistical expertise. FT and CNM: significant manuscript writer. FJT, CNM, CPM, JFR, MJV, VHT, JF, RG, JP, HF, RC, and BJS: manuscript revising critically for important intellectual content and final approval of the version to be published. All authors contributed to the article and approved the submitted version.

## Funding

The publishing cost of this work was supported by the Interdisciplinary Center for the Study of Human Performance—CIPER (Unit 447) under grant UIDB/00447/2020.

## Conflict of Interest

FJT, CNM, JF, and RG are currently employees of a biotechnology company (Bettery S.A.) that produces dietary supplements. This company also developed the plant-based protein matrix (BETTPRO^®^) used in this study. BJS formerly served on the scientific advisory board of Dymatize Nutrition, a manufacturer of sports supplements. HF is employed by José de Mello Saúde. RC, VHT are advisors for Futebol Clube do Porto. The remaining authors declare that the research was conducted in the absence of any commercial or financial relationships that could be construed as a potential conflict of interest.

## Publisher's Note

All claims expressed in this article are solely those of the authors and do not necessarily represent those of their affiliated organizations, or those of the publisher, the editors and the reviewers. Any product that may be evaluated in this article, or claim that may be made by its manufacturer, is not guaranteed or endorsed by the publisher.
